# Investigation into cardiac sympathetic innervation during the commencement of haemodialysis in patients with chronic kidney disease

**DOI:** 10.1186/s41747-017-0027-0

**Published:** 2017-12-06

**Authors:** Walter Noordzij, Akin Özyilmaz, Andor W. J. M. Glaudemans, René A. Tio, Esther R. Goet, Casper F. M. Franssen, Riemer H. J. A. Slart

**Affiliations:** 1 Department of Nuclear Medicine and Molecular Imaging, University of Groningen, University Medical Center Groningen, Groningen, The Netherlands; 2 Division of Nephrology, Department of Internal Medicine, University of Groningen, University Medical Center Groningen, PO Box 30.001, 9700 RB Groningen, The Netherlands; 3 Dialysis Center Groningen, University of Groningen, University Medical Center Groningen, PO Box 30.001, 9700 RB Groningen, The Netherlands; 4 Department of Cardiology, University of Groningen, University Medical Center Groningen, PO Box 30.001, 9700 RB Groningen, The Netherlands

**Keywords:** Chronic kidney disease, Haemodialysis, Myocardial ischaemia, Myocardial scintigraphy, Sympathetic innervation

## Abstract

**Background:**

Patients with chronic kidney disease (CKD) who undergo chronic haemodialysis (HD) show altered sympathetic tone, which is related to a higher cardiovascular mortality. The purpose of this study was to investigate the effect of transition from pre-HD to HD on cardiac sympathetic innervation.

**Methods:**

Eighteen patients aged 58 ± 18 years (mean ± standard deviation [SD]), 13 males and five females, with stage 5 CKD and nine healthy control subjects aged 52 ± 17 (mean ± SD), three males and six females, were included in this prospective study between May 2010 and December 2013. All patients underwent ^123^I-labelled meta-iodobenzylguanidine (^123^I-MIBG) scintigraphy for cardiac sympathetic innervation and electrocardiographically gated adenosine stress and rest ^99m^Tc-labelled tetrofosmin single-photon emission computed tomography for myocardial perfusion imaging prior to (pre-HD) and 6 months after the start of HD. Results of ^123^I-MIBG scans in patients were compared to controls. Impaired cardiac sympathetic innervation was defined as late heart-to-mediastinum ratio (HMR) < 2.0.

**Results:**

Mean late HMR was lower in patients during HD (2.3) than in controls (2.9) (*p* = 0.035); however, in patients it did not differ between pre-HD and after the start of HD. During HD, two patients showed new sympathetic innervation abnormalities, and in three patients innervation abnormalities seemed to coincide with myocardial perfusion abnormalities.

**Conclusions:**

CKD patients show cardiac sympathetic innervation abnormalities, which do not seem to progress during the maintenance HD. The relationship between sympathetic innervation abnormalities and myocardial perfusion abnormalities in HD patients needs further exploration.

## Key points

- End-stage renal disease patients on maintenance haemodialysis have lower mean late heart-to-mediastinum ratio on ^123^I-labelled meta-iodobenzylguanidine scintigraphy than healthy control subjects
- Mean late heart-to-mediastinum ratio does not seem to worsen during the commencement of maintenance haemodialysis
- Patients with signs of cardiac sympathetic innervation abnormalities before the start of haemodialysis developed alterations in myocardial perfusion during haemodialysis

## Background

Chronic kidney disease (CKD) patients who undergo haemodialysis (HD) show increased sympathetic tone and increased serum norepinephrine levels [1–3]. In addition, myocardial blood flow decreases significantly during HD, indicating that acute HD-associated factors play a role in myocardial perfusion abnormalities [4, 5]. In normal population, cardiac ischaemia and autonomic dysfunction are reliable predictors for future cardiac events and are also associated with increased long-term mortality [6–8]. The presence of these findings in CKD patients is related to a higher cardiovascular mortality, exceeding that of age- and gender-matched healthy control subjects [9].

An unknown number of individuals with CKD are completely asymptomatic for coronary artery disease (CAD). Especially CKD patients with concomitant diabetes show silent myocardial ischaemia and diabetic autonomic neuropathy. However, both the extent of cardiac ischaemia and sympathetic innervation abnormalities as well as the timepoint of their development during or even before starting HD are unclear. It was previously shown that cardiomyocytes are more vulnerable to sympathetic innervation abnormalities than to ischaemia, which may indicate that cardiac sympathetic innervation abnormalities precede myocardial ischaemia, [10]. Autonomic dysfunction is also commonly present in end-stage renal disease [11], but whether this results in lack of symptoms in cardiac ischaemia is unknown.

Thus, CKD patients undergoing HD show sympathetic innervation abnormalities. At present, it is unclear whether these abnormalities are present before the start of HD or occur during the commencement of maintenance HD. So far, the presence and severity of cardiac sympathetic innervation abnormalities and CAD has never been studied in asymptomatic end-stage (stage 5) CKD patients who are not yet on HD. Therefore, the aim of this study is to determine baseline cardiac sympathetic innervation in asymptomatic stage 5 CKD patients, and to investigate the early effect of the initiation of maintenance HD on cardiac sympathetic innervation.

## Methods

### Patients

This study was approved by the institutional ethics review board (‘medisch ethische toetsingscommissie’ of the University Medical Center Groningen, chaired by professor WA Kamps, protocol number 2008/133, date of approval June 1, 2008). All included patients gave their written informed consent for participation in this study. Patient information was anonymised and de-identified before data analysis.

Between May 2010 and December 2013 a total of 18 consecutive patients (13 males and five females) with stage 5 CKD were prospectively included. Diagnosis of CKD was based on serum levels of creatinine, and subsequent estimated glomerular filtration rate (eGFR), the latter determined by the Modification of Diet in Renal Disease method [12]. Included were patients aged > 18 years who had no history of CAD and were asymptomatic for present CAD, had no history of Parkinson’s disease or dementia with Lewy bodies, were not currently using tricyclic antidepressant agents which could interfere with ^123^I-labelled meta-iodobenzylguanidine (^123^I-MIBG) uptake, and were expected to start HD within the next 6 months. All patients underwent laboratory tests at baseline. Electrocardiograms in pre-HD and during chronic HD were retrieved from the digital patient chart.

^123^I-MIBG scintigraphy for cardiac sympathetic innervation and ^99m^Tc-tetrofosmin scintigraphy for myocardial perfusion imaging were performed before the start of HD and 6 months after the start of HD. Patients who showed CAD in pre-HD were not excluded from the study to investigate the progression of CAD during maintenance HD. All scans were performed on interdialytic days: a non-weekend day before or after an HD day. Consensus reading on myocardial perfusion scans was performed during general patient care board meetings. All scans were reviewed by an experienced nuclear medicine physician (RHJAS, with > 20 years experience), and cardiologist (RAT, with > 20 years experience).

For ^123^I-MIBG imaging, nine age-matched normal volunteers were scanned, each on one occasion, to collect a healthy control database. All subjects were in good health, i.e. they did not suffer from known (current or previously treated) hypertension, heart disease, diabetes mellitus, or renal impairment and did not use medication.

### Haemodialysis

Patients were dialyzed three times per week. All patients were on bicarbonate dialysis with a low-flux polysulfone hollow-fiber dialyser F8 (Fresenius Medical Care, Bad Hamburg, Germany). Blood flow and dialysate flow rates were 250–350 ml/min and 500 ml/min, respectively. Dialysate temperature was 36.0 or 36.5 °C. Dialysate composition was sodium 139 mmol/l, potassium 1.0 or 2.0 mmol/l, calcium 1.5 mmol/l, magnesium 0.5 mmol/l, chloride 108 mmol/l, bicarbonate 34 mmol/l, acetate 3.0 mmol/l and glucose 1.0 g/dl.

### ^123^I-MIBG scintigraphy

Scintigraphy was performed after blockade of thyroid uptake of free ^123^I by iodine-potassium iodide (Lugol’s solution). After a 15-min resting period, subjects were injected with 185 MBq (5 mCi) ^123^I-MIBG (General Electric Healthcare Medical Diagnostics, Eindhoven, The Netherlands) by an intravenous catheter. At 15 min (early image) and 4 h (late image) after tracer administration, a 10-min static anterior view of the chest was acquired using a Symbia S gamma camera (Siemens Medical System, Knoxville, Tennessee, USA) with a medium-energy low-penetration parallel-hole collimator [13]. A 15% energy window centred on 159 keV, a 256 × 256 matrix size and a 1.45 zoom factor were used. According to guidelines for ^123^I-MIBG scintigraphy, the use of β-adrenoceptor blocking agents (β-blockers) was not discontinued [13].

For planar images, left ventricle (LV) activity was measured over the raw static image using a region of interest along the contour of the LV. A second region of interest was placed over the upper mediastinum [13]. The heart-to-mediastinum ratio (HMR) was measured three times, and the average of measurements was taken into account. Late HMR < 2.0 was considered as a sign of cardiac sympathetic innervation abnormalities [13]. Data were also compared with our local normal database.

### ^99m^Tc-tetrofosmin SPECT

Adenosine stress ^99m^Tc-tetrofosmin (250 MBq, 6.8 mCi) and rest ^99m^Tc-tetrofosmin (750 MBq, 20 mCi) electrocardiographically gated single-photon emission computed tomography (SPECT) were performed in consecutive order in a 1-day protocol to analyse myocardial ischaemia. Patients were asked to withdraw caffeine-containing beverages and/or food 24 h before the examination. ^99m^Tc-tetrofosmin SPECT studies were obtained 1 h after tracer administration using a dual-headed gamma camera, equipped with low-energy high-resolution collimators (Symbia T16 gamma camera, Siemens Medical System, Knoxville, Tennessee, USA), electrocardiographic gating, and low-dose computed tomography for attenuation correction. All data from the ^99m^Tc-tetrofosmin SPECT studies were reformatted to obtain short-axis, horizontal and vertical long-axis sections. Data were analyzed and displayed in a 17-segment polar map using the Quantitative Perfusion SPECT application, a commercially available gated cardiac software package developed by the Cedars-Sinai Medical Center (Los Angeles, CA, USA) [14]. Average counts per segment were obtained from the 17 segments and the measured counts were normalized to the segment with the highest average counts.

### Statistical analysis

Baseline descriptive statistics are presented as mean ± standard deviation or median (range) for continuous variables and numbers with percentages for categorical variables as required. We evaluated differences between the two study groups using the χ^2^ test and Fisher exact test for categorical data and the Student *t*-test and Mann–Whitney *U* test for continuous data, according to whether data were normally distributed. Furthermore, a two-way ANOVA with Bonferroni correction for multiple comparison was performed to analyse the differences in study groups over time. In all analyses, *p* < 0.050 was considered statistically significant. Statistical analysis was performed using the SPSS package version 22 (IBM Corp., Armonk, NY, USA).

## Results

### Patient characteristics

Baseline characteristics of the patients are summarized in Table 1. The mean age was 58 ± 18 years. For the nine healthy controls (three males and six females), the age (52 ± 17 years) was not significantly different (*p* = 0.423). As expected in patients with stage 5 CKD, eGFR indicated severely reduced kidney function (< 15 ml/min) in all patients. A majority of the patients had hypertension (78%) and used anti-hypertensive medication, mainly β-blockers and diuretics. Diabetes mellitus was present in 33% of the patients.

**Table 1:** Patient characteristics at baseline

	Frequency, median (range) or mean ± standard deviation
Gender (number)	
Male	13 (72%)
Female	5 (28%)
Age (years)	58 ± 18
Medical history (number)	
Hypertension	14 (78%)
Hypercholesterolemia	6 (33%)
Diabetes mellitus	6 (33%)
Smoking	2 (11%)
Coronary artery disease	0 (0%)
Medication at baseline (number)	
β-blockers	11 (61%)
ACE ihibitors	6 (33%)
Diuretics	8 (44%)
Laboratory at inclusion	
Leucocytes (× 10^9^/l)	7.1 (3.3–9.8)
Haemoglobin (mmol/l)	6.8 (6.0–9.7)
Trombocytes (× 10^9^/l)	208 (117–347)
C-reactive protein (mg/l)	0 (0–59)
Sodium (mmol/l)	140 (135–145)
Potassium (mmol/l)	4.9 (4.0–5.9)
Chloride (mmol/l)	107 (98.0–114)
Urea (mmol/l)	26 (15–38)
Creatinine (μmol/l)	584 (339–1.31 × 10^3^)
eGFR (ml/min × 1.73 m^2^)	8.0 (4.0–12)
Uric acid (mmol/l)	0.45 (0.23–0.72)
Calcium (mmol/l)	2.25 (2.07–2.53)
Phosphate (mmol/l)	1.60 (1.07–2.12)
Total protein (g/l)	67 (60–77)
Albumin (g/l)	41 (29–46)
High-density lipoprotein cholesterol (mmol/l)	1.0 (0.70–2.7)
Low-density lipoprotein cholesterol (mmol/l)	2.2 (0.80–4.8)
Glucose (mmol/l)	6.7 (4.1–14)
Urinary protein excretion (g/24 h)	2.7 (0.40–6.8)

*ACE* angiotensin converting enzyme, *eGFR* estimated glomerular filtration rate

At baseline, none of the patients had electrocardiographic signs of ischaemia or infarction. One patient had a first degree atrio-venticular block whereas another patient had a left bundle branch block (QRS duration 138 ms).

### ^123^I-MIBG scintigraphy

**Fig. 1:**
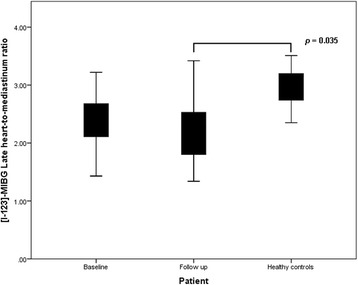
Late ^123^I-MIBG heart-to-mediastinum ratio (HMR) in pre-HD and during chronic HD, and of healthy control subjects. Late HMR was significantly lower in patients during chronic HD than in healthy controls

Table 2 shows the results of ^123^I-MIBG scans. All 18 patients underwent ^123^I-MIBG scintigraphy in pre-HD and during chronic HD. ^123^I-MIBG scans during chronic HD were performed within 6 ± 2 months (range 3–13 months) after the start of HD. Late HMR in patients in pre-HD was not different from healthy controls. There was no significant difference (especially no significant decrease) in late HMR between pre-HD and chronic HD. However, during chronic HD patients showed significant lower late HMR than healthy controls: mean late HMR 2.3 vs 2.9 (*p* = 0.036; Fig. 1). In pre-HD, three patients had signs of impaired cardiac sympathetic innervation, whereas five patients showed cardiac sympathetic innervation abnormalities during chronic HD. None of these five patients used angiotensin converting enzyme inhibitors. Four of these patients did use β-blockers. Only one patient with diabetes mellitus developed cardiac sympathetic innervation abnormalities during HD.

**Table 2:** ^123^I-MIBG findings

	Frequency or mean ± standard deviation		
	Controls (n = 9)	Patients	
		Pre-HD (n = 18)	Chronic HD (n = 18)
Late heart-to-mediastinum ratio (HMR)	2.9 ± 0.58	2.4 ± 0.76	2.3 ± 0.64
Positive for cardiac sympathetic innervation abnormalities^a^	0	4	5

*HD* haemodialysis, *n* number

^a^Patients with low late HMR and/or high washout rate

Late HMR at-follow up was significantly different from controls (*p* = 0.035; 95% confidence interval of the difference −1.1, −0.46)

### ^99m^Tc-tetrofosmin SPECT

The results of myocardial perfusion SPECT are summarized in Table 3. Pre-HD myocardial perfusion SPECT was performed in 17 out of 18 (94%) patients. One patient was not able to undergo the investigation before the start of HD. Myocardial ischaemia was present in six (35%) patients, and five of these six patients also showed wall motion abnormalities, especially hypokinesia of the affected LV wall. None of the patients had signs of infarction in pre-HD. Mean LV ejection fraction (56 ± 17%) was within a normal range.

**Table 3:** Gated myocardial perfusion SPECT results

	Frequency or mean ± standard deviation	
	Baseline (n = 17)	Follow up (n = 17)
End-diastolic volume (ml)	137 ± 41.2	122 ± 53.2
Left ventricular ejection fraction (%)	56 ± 7.0	58 ± 7.0
ischaemia	6	4
Infarction	0	2

*n* number

Chronic HD myocardial perfusion SPECT was performed within 6 ± 2 months (range 3–13 months) after the start of HD. During chronic HD, four patients showed myocardial ischaemia. Two of these four patients had developed new myocardial ischaemia during HD while in the other two patients myocardial ischaemia was already present in pre-HD. One of these latter two patients was an elderly lady with a prolonged history of diabetes mellitus. Two patients with myocardial ischaemia in pre-HD had normal myocardial perfusion SPECT results during chronic HD. Both patients with myocardial infarction during chronic HD showed progression from myocardial ischaemia in pre-HD.

During chronic HD, neither mean LV ejection fraction nor mean end-diastolic volumes were statistically different from pre-HD.

### Relationship between myocardial perfusion and sympathetic innervation

**Fig. 2:**
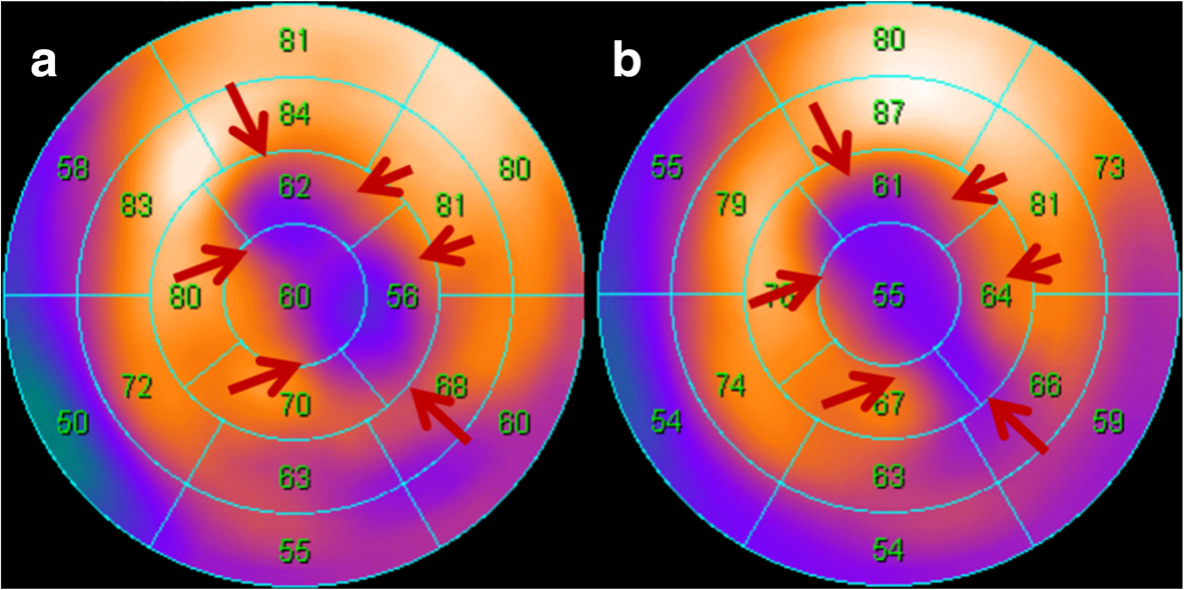
Polar map reconstruction of the myocardial perfusion SPECT of a patient during chronic HD: **a** stress image; **b** rest image. The persisting perfusion defect in the antero-apical segments of the left ventricle wall (*red arrows*) indicates myocardial infarction

**Fig. 3:**
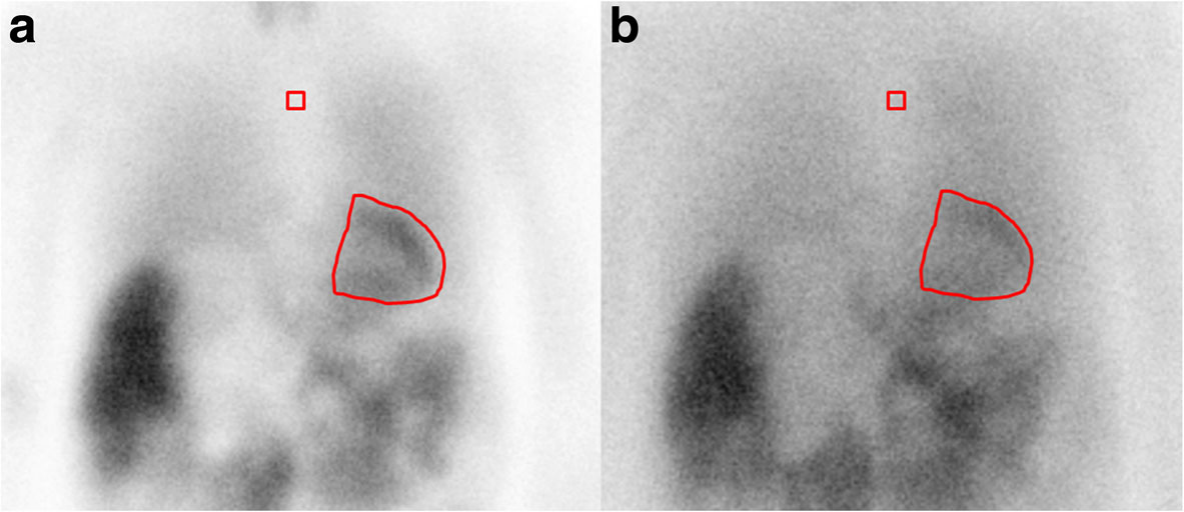
Planar images from ^123^I-MIBG scintigraphy of a patient during chronic HD. **a** Early image 15 min after tracer administration; **b** late image 4 h after tracer administration. Late heart-to-mediastinum ratio (HMR) is the ratio between the counts within a reference point in the upper mediastinum (*red square box*) and the left ventricle (*red contoured region*). The HMR of panel **a** was 3.03 and of panel **b** 1.80, resulting in a 41% washout

Four patients already had signs of cardiac sympathetic innervation abnormalities in pre-HD. One patient with a positive ^123^I-MIBG scan during chronic HD developed new myocardial ischaemia, one patient developed an infarction out of ischaemia in pre-HD (Figs. 2 and 3) and one patient showed an increase in ischaemic area. Two patients with positive ^123^I-MIBG scans during chronic HD had normal myocardial perfusion scans in pre-HD and during chronic HD. These two patients, who showed normalization of myocardial ischaemia in pre-HD, had no signs of cardiac sympathetic innervation abnormalities in pre-HD or during chronic HD.

## Discussion

During the commencement of HD, mean late HMR was lower than in healthy controls but not compared to patients in pre-HD. The presence of cardiac sympathetic innervation abnormalities detected by ^123^I-MIBG scintigraphy in HD patients is not a novel concept. However, a statistical difference in HMR between patients and healthy controls has never been established before [15, 16], nor was it in this study. In addition, a difference in late HMR between baseline and during the commencement of maintenance HD could not be established. A previous study showed that HMR is lower in patients undergoing maintenance HD for a prolonged period of time, up to 120 months [17]. However, in that study ^123^I-MIBG scintigraphy was performed at only one single time point: the effect on HMR of transition from pre-HD to HD was not investigated. The present study provides additional information that cardiac sympathetic innervation abnormalities do not seem to worsen during the commencement of maintenance HD.

The results of this study should be interpreted with caution due to limited power. A power analysis was performed and resulted in a required number of 44 included patients. Initially, patients with CAD detected on pre-HD myocardial perfusion SPECT were excluded from this study. Due to a very slow inclusion of patients with no CAD at all, we decided to also include patients with CAD in pre-HD. This did not result in an increase in the inclusion rate. Therefore, the study was terminated before the necessary number of patients was included. Eventually, only 18 patients completed the study protocol. If the required number of patients had been included, the results might have been more definitive. However, the present study comprises the largest group of patients in which cardiac sympathetic innervation has been evaluated during the initiation of maintenance HD.

In addition, the development of myocardial perfusion abnormalities during the commencement of maintenance HD should be interpreted with caution. Patients who showed myocardial ischaemia in pre-HD were treated with maximal anti-ischaemic medication. The affected areas were too small to refer for percutaneous intervention. We cannot be sure that treatment influenced the outcome of the scans during chronic HD. Since two out of the three patients with low late HMR in pre-HD showed alteration in myocardial perfusion from pre-HD to chronic HD, this may suggest that cardiac sympathetic denervation precedes the development of myocardial perfusion defects. This suggestion supports the fact that cardiomyocytes are more susceptible to sympathetic innervation abnormalities than to decreased blood flow [18]. Furthermore, two patients in this study also developed sympathetic denervation without myocardial perfusion abnormalities, which may suggest (not yet detectable) microvascular coronary dysfunction. However, six of the included 18 patients already showed myocardial ischaemia in pre-HD despite being asymptomatic for CAD. Then again, with the reported small areas of myocardial ischaemia and infarction in this cohort consisting of asymptomatic patients, the detection rate of myocardial perfusion SPECT was rather limited. Furthermore, cardiac magnetic resonance imaging seems to outperform SPECT for the detection of CAD [19]. The reliability of the scans of the affected ischaemic or infarcted areas could also have been improved by performing inter-observer agreements. Although experienced physicians reviewed the myocardial perfusion SPECT scans in general patient care board meetings, this consensus reading is not a substitute for inter-observer agreement analysis.

Other important issues that influence outcome are the time between baseline scans and the start of HD as well as the time between the start of HD and follow-up scans. It appeared to be very difficult to determine the time point for imaging, particularly in patients with very fragile kidney function. Some of the patients had stable CKD for a prolonged period of time but underwent sudden and rapid deterioration. There were also patients who required the start of HD to be postponed because the deterioration in kidney function was not as rapid as expected. The largest time frame between baseline to follow-up scans was 13 months. Unfortunately, the researchers were not always aware of this change in schedule. Therefore, uncertainty about the start of HD resulted in the differences in the time-interval of imaging.

According to the 2012 United States Renal Data System annual report, arrhythmia and sudden cardiac arrest accounted for 63% of the cardiovascular and 27% of all-cause mortality in dialysis patients [20]. In a recent study involving 75 HD patients with an implantable cardioverter defibrillator (ICD), nearly 80% of all sudden cardiac arrests were caused by ventricular tachycardia and ventricular fibrillation [21]. The authors reported a higher survival rate after sudden cardiac arrest than previously assumed. ^123^I-MIBG scans are known to have predictive implications for successful ICD therapy in patients with heart failure [22]. Therefore, ^123^I-MIBG scintigraphy has the potential to play a guiding role in clinical ICD decision-making in HD patients. Until that time, prospective studies have to demonstrate the relationship between low late HMR and the development of ischemic heart failure-related arrhythmia during HD.

To summarise, stage 5 CKD patients on maintenance HD showed a lower mean late HMR than healthy controls. Mean late HMR does not seem to worsen after the commencement of HD or during maintenance HD. Although present in a minority of the patients, signs of cardiac sympathetic innervation abnormalities in pre-HD may lead to alterations in myocardial perfusion during chronic HD. Further prospective studies should provide more insight into the long-term effect of cardiac sympathetic innervation abnormalities on the development of ischaemic heart failure-related arrhythmia during HD.

## Abbreviations

ACE: Angiotensin converting enzyme
CAD: Coronary artery disease
CKD: Chronic kidney disease
eGFR: Estimated glomerular filtration rate
HD: Haemodialysis
HMR: Heart-to-mediastinum ratio
ICD: implantable cardioverter defibrillator
LV: Left ventricle
MIBG: meta-iodobenzylguanidine
SD: Standard deviation
SPECT: Single-photon emission computed tomography
